# Facial attractiveness in the eyes of men with high arousal

**DOI:** 10.1002/brb3.3132

**Published:** 2023-06-27

**Authors:** Shangfeng Han, Jie Gao, Wenjuan Xing, Xinyi Zhou, Yuejia Luo

**Affiliations:** ^1^ Department of Psychology and Center for Brain and Cognitive Sciences, School of Education Guangzhou University Guangzhou China; ^2^ Shenzhen Key Laboratory of Affective and Social Neuroscience, Center for Brain Disorders and Cognitive Sciences, School of Psychology Shenzhen University Shenzhen China; ^3^ School of Psychology, Sichuan Center of Applied Psychology Chengdu Medical College Chengdu China; ^4^ College of Economics and Management Qilu Normal University Jining China; ^5^ Institute for Neuropsychological Rehabilitation University of Health and Rehabilitation Sciences Qingdao China; ^6^ The State Key Lab of Cognitive and Learning, State Key Laboratory of Cognitive Neuroscience and Learning, Faculty of Psychology Beijing Normal University Beijing China

**Keywords:** alpha, arousal, facial attractiveness, gender difference

## Abstract

**Introduction:**

Individuals differ in how they judge facial attractiveness. However, little is known about the role of arousal level and gender differences in individuals’ facial attractiveness judgments.

**Methods:**

We used resting‐state electroencephalogram (EEG) to investigate this issue. A total of 48 men (aged 22.5 ± 3.03 years [mean ± *SD*], range: 18–30 years) and 27 women (aged 20.3 ± 2.03 years [mean ± *SD*], range: 18–25 years) participated in the experiment. After the EEG was collected, participants were instructed to complete a facial attractiveness judgment task. Connectome‐based predictive modeling was used to predict individual judgment of facial attractiveness.

**Results:**

Men with high arousal judged female faces as more attractive (*M* = 3.85, *SE* = 0.81) than did men with low arousal (*M* = 3.33, *SE* = 0.81) and women (*M* = 3.24, *SE* = 1.02). Functional connectivity of the alpha band predicted judgment of female facial attractiveness in men but not in women. After controlling for the age and variability, the prediction effect was still significant.

**Conclusion:**

Our results provide neural evidence for the enhancement of the judgment of facial attractiveness in men with high arousal levels, which supports the hypothesis that individuals’ spontaneous arousal contributes to variations in facial attractiveness preferences.

## Graphical abstract

Our findings supported the hypothesis pertaining to the general arousal mechanism. Our study contributes important insights into the link between individual spontaneous brain activation and social preference.

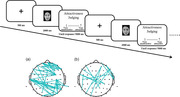

## 1 INTRODUCTION

Facial attractiveness is a key factor in facial impression (Todorov et al., 2015), which influences decisions, particularly mate choice (Buss & Schmitt, 2019; Pandey & Zayas, 2021). Highly attractive faces are associated with greater benefits. For example, individuals with attractive faces are considered healthy and as having good traits (Mengelkoch et al., 2022; Tsukiura & Cabeza, 2011). The judgment of facial attractiveness varies in different individuals (Han, Liu, et al., 2020; Little, 2014; Thiruchselvam et al., 2016). Brain functional connectivity is an objective index that can predict human behavior (Shen et al., 2017). However, the role of individual spontaneous activation of the brain (such as arousal) in the judgment of facial attractiveness remains unclear.

Arousal plays an important role in attractiveness processing (Montoya & Horton, 2020). A previous study found that high‐arousal state evoked by a swaying bridge enhanced attractiveness ratings compared with the low‐arousal state evoked by a stable bridge (Dutton & Aron, 1974 ). Recently, researchers used the pheromone androstenol to evoke a high‐arousal state that contributed to participants rating faces as significantly more attractive compared with the control odor (Beaton et al., 2022). The theory of misattribution of arousal proposes that a state of high arousal causes individuals to misattribute this feeling to attraction (Little, 2014; White et al., 1981). Arousal has also been interpreted according to the context. For example, enhanced arousal was suggested to lead to polarize outcomes in the negotiation context (Brown & Curhan, 2013). In this study, the theory of misattribution of arousal was extended by focusing on the spontaneous arousal of the perceiver. This extension is meaningful because individual differences exist in the influence of context‐evoked arousal on the judgment of attractiveness (Jouffre, 2015). Furthermore, the arousal level was rated subjectively in previous studies (Fayolle et al., 2015; Zhou et al., 2021). However, a resting‐state electroencephalography (EEG), an objective approach, was applied in this study to measure individual spontaneous arousal. Alpha power in the resting state is the classic index of arousal, with a smaller alpha reflecting stronger arousal (Barry et al., 2020; Pivik & Harman, 1995). Therefore, the EEG method was deemed suitable to investigate the influence of individual arousal on the perception of facial attractiveness.

Gender differences exist in the processing of facial attractiveness. To cope with sex‐specific adaptive problems, in the mate choice, men place greater value on female attractiveness: an attractive female face signals a potential mating opportunity for men (Buss & Schmitt, 2019). Men also show stronger activation of the area of the brain associated with reward when judging facial attractiveness than women (Cloutier et al., 2008). Men are able to quickly process female faces, and the effect was not influenced by the difficulty of task, which suggests that male process female faces automatically (Klümper et al., 2020). Conversely, an attractive female face poses a potential threat to other women's reproductive success (Buss & Schmitt, 2019). For this reason, women prefer fewer female applicants because of intrasexual competition (Luxen & Vijver, 2006). Furthermore, one study found that women's arousal was unrelated to their male facial attractiveness judgment (Hagerman et al., 2017), and others determined that when using music to evoke arousal, women but not men judged male faces as more attractive than in the silent control condition (Marin & Rathgeber, 2022; Marin et al., 2017). Women rate male faces as significantly less attractive than men rate female faces (Fisher, 2004). Additionally, previous studies have found some meaningful results concerning women rating male facial attractiveness. Bech‐Sørensen and Pollet (2016) showed that women emphasized facial attractiveness less than men, valued a high earning potential more than men did, and had a greater preference for marrying someone older. The sexual strategy theory also suggests that men and women have evolved to pursue different mating strategies, with men being more attentive to facial attractiveness, while women value characteristics signaling resources (e.g., power, status; Buss & Schmitt, 2019), which are rarely reflected in faces. Therefore, the female face was considered the target in this study.

To further determine the association between the brain and judgment of facial attractiveness, we employed connectome‐based predictive modeling (CPM), which has been widely applied to the prediction of individual behavior (Cai et al., 2020). CPM aims to identify functional connections throughout the brain that can predict a specific behavioral measure based on individual differences in connectivity strength. One important feature of CPM is that a set of functional connections, recognized as a predictor of a behavioral variable in one sample, can effectively generalize the same behavioral variables in independent samples. By using CPM, researchers have successfully predicted individual childhood aggression (Ibrahim et al., 2022), processing speed in older adults (Gao, 2020), and loneliness (Feng et al., 2019), identifying related biomarkers. However, an EEG biomarker that can predict individual facial attractiveness preference remains unknown.

In summary, we aimed to explore the influence of spontaneous arousal on individual judgment of facial attractiveness and gender differences. Since women value male resources more than facial attractiveness in mate selection (Buss & Schmitt, 2019), only female faces were used as the target faces in the current study. Men (mate choice) and women (intrasexual competition) were instructed to judge the attractiveness of female faces. Resting‐state EEGs were also collected. We expected that men with high arousal levels would judge female faces as more attractive than would those with low arousal levels (H1). Because of gender differences, an attractive female face signals mate choice in men but intrasexual competition in women (Luxen & Vijver, 2006). Thus, we expected that men would give higher scores to female faces than women would (H2). CPM was used to identify the individual preference in the judgment of facial attractiveness. As previously mentioned, arousal is strongly associated with attractiveness, particularly for men in terms of judging women's faces (Little, 2014). Thus, we expected that the functional connectivity of the alpha band could predict men's perception of female attractiveness (H3).

## 2 MATERIALS AND METHODS

### 2.1 Participants

Eighty participants were recruited from a university. Two men and three women were excluded due to excessive EEG artifacts (an EEG amplitude that was greater than 100 μV for more than half of the total time). Finally, 48 men (aged 22.5 ± 3.03 years [mean ± *SD*], range: 18−30 years) and 27 women (20.3 ± 2.03 years [mean ± *SD*], range: aged 18−25 years) with no history of psychiatric or neurological diseases completed the experiment. All participants were heterosexual, were right‐handed, and had normal or corrected vision. All participants provided informed consent. This study was conducted in accordance with the Declaration of Helsinki and approved by a local ethics committee. All participants received monetary compensation of 40 RMB after completing the experiment.

### 2.2 Materials

A face dataset of 180 Chinese young female photos was downloaded from the Internet. In the photos, the hair and other accessories on the faces were removed and erased using Adobe Photoshop CS 2014. Internal features of the faces were retained, and all images were adjusted to black‐and‐white with consistent gray, size, contrast, and brightness. These pictures were scored by 30 participants (aged 18−26 years; 50% men) on a 7‐point Likert scale using an online survey. Each picture took the average of all scores for each participant, with scores arranged from the lowest to the highest. Eighty middle‐attractive female faces were selected (*M* = 3.28, *SD* = 0.18), which were located from the 27th percentile to the 73rd percentile of all photos used as experimental materials. The average age of the women in the pictures was 23.8 (*SD* = 0.26).

### 2.3 Procedure

Resting‐state EEG data for 200 s were recorded for each participant. The participants sat in a chair and were instructed to maintain a comfortable posture, close their eyes but remain awake, and refrain from moving.

**FIGURE 1:**
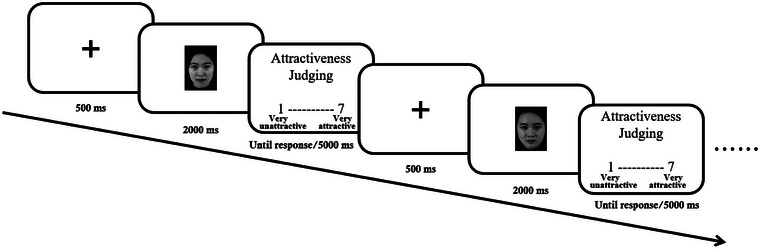
The schematic of a trial. The fixation cross, a female face, and Likert‐type scale would be presented in sequence.

After resting‐state EEG acquisition, participants performed the experimental tasks. The procedure of this experiment was presented using E‐Prime software (version 3.0; Figure 1). Each trial was preceded by a fixation cross in the center of the screen for 500 ms, followed by a 2000‐ms female face image at random. A schematic of a 7‐point Likert‐type scale (1 = *very unattractive* and 7 = *very attractive*) was then displayed, and participants were instructed to judge facial attractiveness by pressing a number from 1 to 7 on a keyboard. The schematic scale disappeared when the participants did not press the key within 5000 ms. A total of 80 trials were conducted in the formal experiment.

### 2.4 EEG data recording and processing

EEG data were recorded at a sample rate of 500 Hz, with electrode caps containing 64 Ag/AgCl electrodes arranged in a 10−20 system and referenced to the midline frontal–central electrode (FCz). Electrode impedance was maintained below 5 kΩ.

EEG data were imported into EEGLAB (Delorme & Makeig, 2004) in MATLAB (Mathworks, v2016a). The processing analysis steps of resting‐state EEG data were as follows: (1) Through visual inspection, bad channels that contained excessive noise or artifacts were marked and interpolated. (2) The EEG data were re‐referenced to an average reference. (3) The EEG data were filtered offline using a band‐pass filter (0.1−30 Hz after a notch filter for 50 Hz line noise). (4) An independent component analysis was used to remove the components related to eye movements, blinks, head movement, electrocardiogram, and other types of artifacts (Delorme et al., 2007). (5) Data were deleted from the analysis if artifacts at the signal channel accounted for more than 50% of the recording time. After preprocessing, behavioral and EEG data of five participants (two men and three women) were excluded from further analysis. (6) Finally, the data were subscribed to the time–frequency analysis and CPM analysis.

### 2.5 Data analysis

A time–frequency analysis was used to quantify neural activity using the FieldTrip toolbox (Oostenveld et al., 2011). An EEG power spectrum was calculated using the fast Fourier method applied on Hanning‐tapered 2‐s windows of the component time courses (1−30 Hz, frequency resolution of 0.5 Hz). Power spectral estimates in the alpha frequency (8−13 Hz bands) were calculated for each participant. The power measurements were averaged across all channels (Härpfer et al., 2021; Knyazev et al., 2004).

We compared the EEG powers of men with high and low arousal in the alpha band. Men were divided into high‐ or low‐arousal groups according to the median alpha power (2.56 dB). The alpha power of men with high arousal (*M* = 1.30, *SD* = 0.63) was significantly lower than that of men with low arousal (*M* = 4.81, *SD* = 3.54) (*t* [48] = −4.78, *p* < .001, *d* = −1.38). To investigate the influence of arousal on the judgment in gender differences, women were also analyzed as a separate group. The alpha power value of women was *M* = 3.25, *SD* = 2.82.

Statistical analyses were performed using SPSS (version 25.0). To determine the influence of arousal on facial attractiveness judgment, a one‐way analysis of variance (ANOVA) was conducted for the facial attractiveness group scores among men with high and low arousal and women in the alpha band. The Bonferroni method was used to adjust for post hoc multiple comparisons.

Following the methods described by Shen et al. (2017), CPM was conducted to predict individual preferences for facial attractiveness. Specifically, the weighted phase lag index (WPLI) was used to calculate resting‐state functional connectivity between all electrodes in each band. WPLI minimizes the effects of volume conduction and reference electrode, which is the delineation of reliable estimators for determining the phase synchronization between two signals (Hardmeier et al., 2014; van Diessen et al., 2015; Vinck et al., 2011). WPLI was first calculated for each pair of electrodes per epoch and then used to generate the average WPLI weight matrix per subject in each band. WPLI was calculated for delta (1−4 Hz), theta (4−7 Hz), alpha (8−13 Hz), and beta (13−30 Hz) bands using a Butterworth band‐pass filter and averaged within each band for each participant. Finally, we obtained a three‐dimensional (channel × channel × participant) functional connection matrix of each band to conduct the CPM.

To identify gender differences in the prediction of facial attractiveness, the functional connection between men and women was used. The CPM was conducted as follows: (1) Leave‐one‐out cross‐validation (LOOCV) was used to divide the training and testing sets; namely, one participant was left out as the testing set, and all other participants were used as the training set. (2) Significant edges were selected. To identify which edges in connection strength can predict judgment of facial attractiveness, we correlated each edge in the connectivity matrix with judgment of facial attractiveness and set the threshold at *p* < .05, resulting in networks composed of edges that were positively and negatively associated with the judgment of facial attractiveness. The positive and negative networks for each participant were summed to identify the combined network. (3) The significant edges were summed for each participant. (4) A predictive model was built. LOOCV was conducted to build linear regressions that model the association between the strength of the positive, negative, and summed networks and the scores of facial attractiveness. (5) The predicted behavioral variable was calculated. The summed edges of each left‐out participant in the test data were inputted into the predictive model to generate the predicted facial attractiveness scores of each participant in the test data. This procedure did not end until each participant obtained the predicted judgment of facial attractiveness. Note that the predicted scores of the positive, negative, and summed networks for each participant were generated using this process. (6) The predictive model was evaluated using the Pearson's correlation coefficient between the predicted and actual values. (7) A significance test was performed; specifically, a permutation test was used to determine the significance of the correlation between predicted and observed values. Judgment of facial attractiveness was randomly shuffled across the participants, and the LOOCV prediction procedure was repeated 1000 times. The *p*‐value was calculated as the number of times the permuted value was greater than the true value and then divided by 1000.

### 2.6 Control and variability analysis

Age was controlled to rule out its potential influence on the predictive model. LOOCV and the premutation test were performed 1000 times and were calculated. To verify the stability of the results, we used thresholds of *p* < .05 and *p* < .01.

## 3 RESULTS

### 3.1 Behavioral results

**FIGURE 2:**
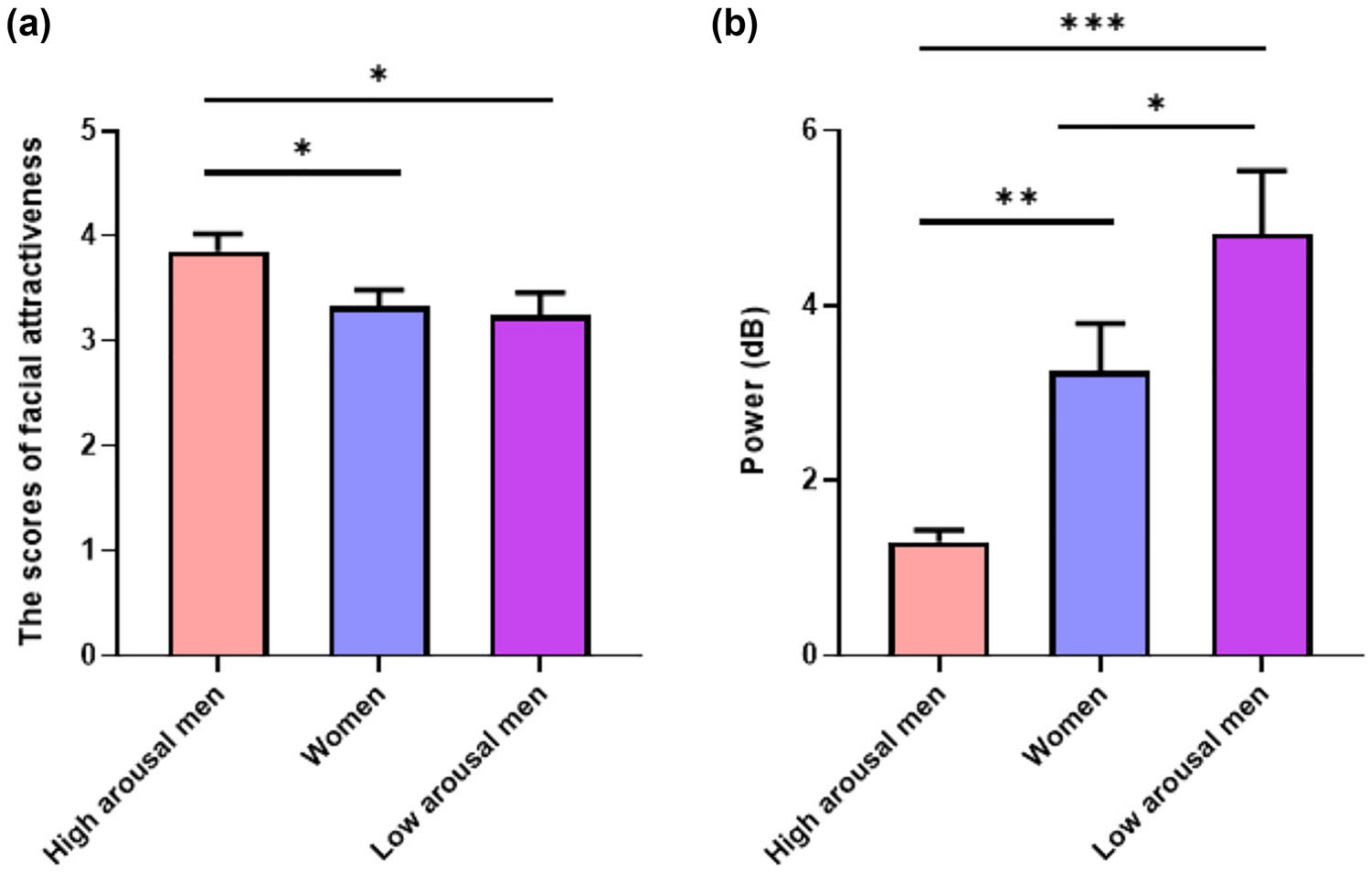
The results of attractiveness judgment and alpha power among high‐ and low‐arousal men and women. *p < .5; **p < .01.

The one‐way ANOVA revealed a significant effect of judgment of facial attractiveness (*F*[2, 72] = 3.30, *p* = .04, *η*_p_^2^ *=* .08). The post hoc analysis showed that the facial attractiveness judgment of men with high arousal (*M* = 3.85, *SE* = 0.81) was significantly higher than that of men with low arousal (*M* = 3.33, *SE* = 0.81) and women (*M* = 3.24, *SE* = 1.02) (all *p* < .04), whereas there was no significant difference between men with low arousal and women (*p* = .74). This indicated that men with high arousal judged female faces to be more attractive. An independent sample *t* test was used to analyze the influence of gender on facial attractiveness judgment, and the result was not significant, *t*(73) = 0.99, *p* = .32 (Figure 2).

### 3.2 EEG results

**FIGURE 3:**
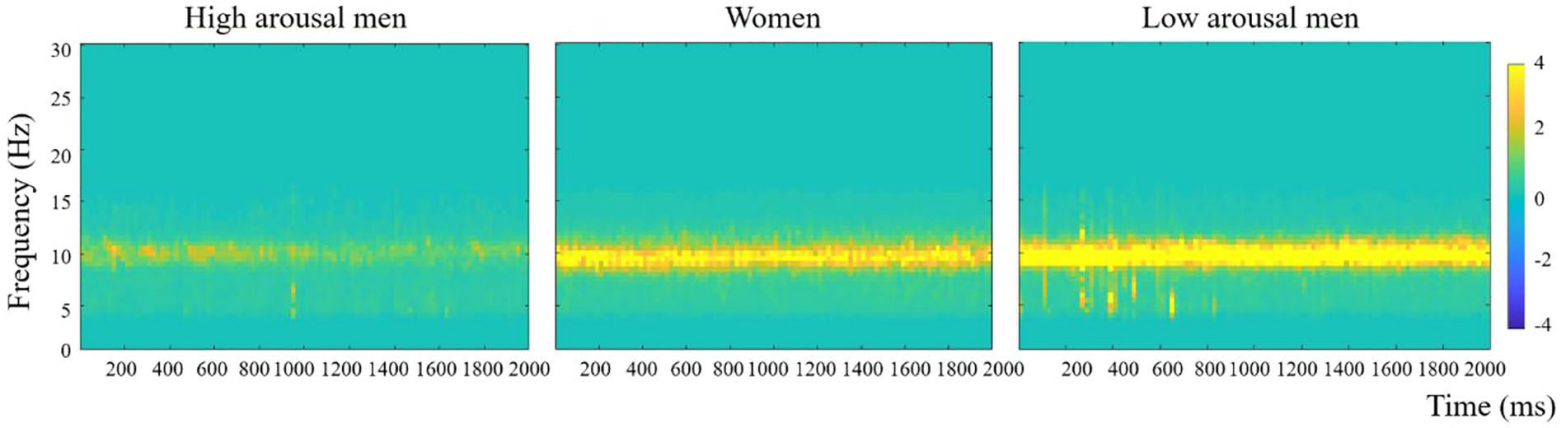
The alpha power of high‐ and low‐arousal men and women evoked during the resting state.

Alpha power was significant among women and men with high and low arousal (*F*[2, 72] = 10.59, *p* < .001, *η*_p_^2^ *=* .23). The alpha power of men with high arousal (*M* = 1.30, *SD* = 0.63) was lower than that of men with low arousal (*M* = 3.25, *SD* = 2.82) and women (*M* = 4.81, *SD* = 3.55) (*p*s < .01). The alpha power of men with low arousal was significantly lower than that of women (*p* = .04; Figure 3). This indicated that the groups had been successfully divided according to arousal.

### 3.3 CPM results

For men, the CPM results revealed that the combined network successfully predicted judgment of facial attractiveness using alpha functional connectivity (*r* = .37, *p* < .01), which remained unchanged after the permutation test (*p*_perm_ = .01). However, the positive (*r* = .20, *p* = .18, *p*_perm_ = .42) and negative networks (*r* = .15, *p* = .32, *p*_perm_ = .48) failed to predict judgment of facial attractiveness. No significant results were found in the prediction models constructed using the other bands in men (see Table 1).

**TABLE 1:** Results of the prediction of the judgement of facial attractiveness.

		Combined network			Positive network			Negative network		
		*r*	*p*	*p*_permu_	*r*	*p*	*p*_permu_	*r*	*p*	*p*_permu_
Men										
	Delta	.10	.52	.27	−.03	.82	.68	.05	.72	.51
	Theta	−.11	.45	.68	–	–	–	.04	.80	.49
	Alpha	.37	<.01^**^	.01^**^	.20	.18	.42	.15	.32	.48
	Beta	.25	.09	.08	.004	.98	.70	.27	.06	.33
Women										
	Delta	.42	.03^*^	.03^*^	.17	.38	.29	.23	.25	.22
	Theta	−.05	.82	.52	.18	.38	.41	−.28	.15	.95
	Alpha	−.47	.47	.60	.04	.86	.76	–	–	–
	Beta	.27	.16	.12	.11	.59	.63	.12	.55	.59

*Note*: Dash (–) indicates that no functional connectivity can predict the behavior performance.

*p < .5; **p < .01.

**FIGURE 4:**
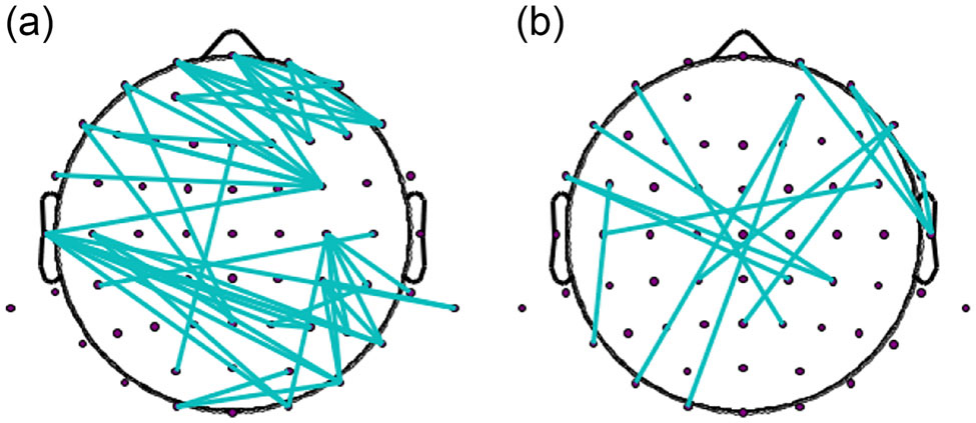
(A) The combined network of the alpha that predicts the behavior performance in men and (B) the combined network of the delta that predicts the behavior performance in women.

**FIGURE 5:**
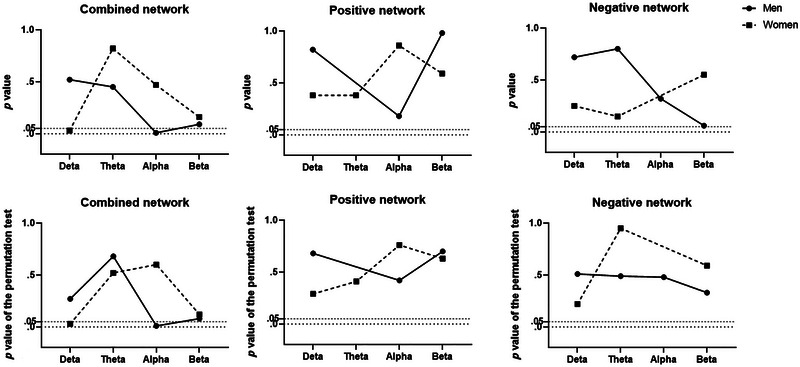
The *p* values without (the top line) and with (the bottom line) permutation test that were calculated by the connectome‐based predictive modeling.

For women, the combined network of the delta band significantly predicted judgment of facial attractiveness, which was also successfully predicted after the premutation test (*r* = .42, *p* = .03, *p*_perm_ = .03). The other bands of the network did not predict the judgment of facial attractiveness (see Table 1; Figures 4 and 5).

### 3.4 Age control

After controlling for age, the alpha functional connectivity in men remained significant for the combined network (*r* = .33, *p* = .02, *p*_perm_ = .03). No significant results were observed in the positive (*r* = .11, *p* = .47, *p*_perm_ = .57) or negative networks (*r* = .16, *p* = .28, *p*_perm_ = .44). No other significant results were observed (see Tables 2 and 3).

**TABLE 2:** Results of control and validation analyses for the prediction of the judgement of facial attractiveness in men.

		Combined network			Positive network			Negative network		
		*r*	*p*	*p*_permu_	*r*	*p*	*p*_permu_	*r*	*p*	*p*_permu_
Age control										
	Delta	−.16	.29	.72	−.05	.72	.72	−.11	.47	.83
	Theta	−.01	.95	.47	–	–	–	.03	.86	.53
	Alpha	.33	.02*	.03*	.11	.47	.57	.16	.28	.44
	Beta	.02	.91	.43	–.01	.93	.73	–.04	.77	.76
Variability results (*p* < .01)										
	Delta	–.26	.07	.82	–.06	.42	.98	–	–	–
	Theta	–.19	.19	.75	–	–	–	–.19	.19	.97
	Alpha	.37	<.01**	.02*	–	–	–	.21	.15	.82
	Beta	–.15	.29	.64	–	–	–	–	–	–

*Note*: Dash (–) indicates that no functional connectivity can predict the behavior performance.

* *p* < .5;

** *p* < .01.

**TABLE 3:** Results of control and validation analyses for the prediction of the judgement of facial attractiveness in women.

		Combined network			Positive network			Negative network		
		*r*	*p*	*p*_permu_	*r*	*p*	*p*_permu_	*r*	*p*	*p*_permu_
Age control										
	Delta	.20	.31	.20	.14	.48	.33	−.01	.96	.58
	Theta	−.08	.71	.53	.13	.52	.44	–.29	.15	.94
	Alpha	−.15	.46	.63	.03	.90	.83	–	–	–
	Beta	.25	.21	.15	.07	.71	.66	.09	.64	.62
Variability results (*p* < .01)										
	Delta	.21	.29	.22	–	–	–	.32	.10	.57
	Theta	.22	.27	.20	–	–	–	.09	.65	.83
	Alpha	−.66	<.01	.80	–	–	–	–	–	–
	Beta	.05	.80	.40	–	–	–	−.05	.81	.97

*Note*: The negative *r* value means failure to predict the behavior performance.

### 3.5 Variability results

When the threshold was set at .01, the combined network of the alpha could predict judgment of attractiveness in men (*r* = .37, *p* < .01, *p*_perm_ = .03). No other significant differences were observed (see Tables 2 and 3).

## 4 DISCUSSION

This study examined the influence of individual arousal on judgment of facial attractiveness and gender differences. We found that men with high arousal judged faces as more attractive than did men with low arousal and women. The arousal index of alpha predicted a judgment of facial attractiveness in men. Overall, these results provide evidence that men with high arousal are associated with higher facial attractiveness judgments. Furthermore, alpha oscillations formed the association between arousal and attractive facial preference. Our results contribute to the understanding of how a positive first impression is formed in facial attractiveness.

The enhancement in facial attractiveness is due to familiarity (Carr et al., 2017), emotion (Han et al., 2022; Han, Liu, et al., 2020), good traits (He et al., 2022), and generalization to similar faces (Han, Hu, et al., 2020). We found the influence of arousal on the judgment of facial attractiveness, namely, men with high arousal rate female faces as more attractive, thereby supporting the misattribution of arousal hypothesis, which suggests individual misattributed arousal due to attractive features (White et al., 1981). The result adds to evidence that individual arousal contributes to improving facial attractiveness. Moreover, the novel finding of the current study is that spontaneous arousal, rather than evoked arousal, is involved in the judgment of facial attractiveness.

Individual differences in attraction preferences also exist among the genders. Men value physical attractiveness of potential partners more than women (Schwarz & Hassebrauck, 2012); thus, they do not require attentional resources when processing female faces but not when processing same‐sex faces. Whether the target face is male or female, women utilize cognitive resources for processing (Klümper et al., 2020). These results are consistent with the evolutionary perspective on facial attractiveness—that is, men easily capture a potential mate's facial attractiveness, and cues of fertility and healthiness, which could increase reproductive success (Buss & Schmitt, 2019). Furthermore, we found that men with high arousal gave higher attractiveness ratings to female faces. Because we used the resting‐state EEG before the participants viewed attractive faces, the enhancement of facial attractiveness may be associated with the general arousal state rather than sexual arousal specifically (Jozifkova & Konvicka, 2009), thus supporting the idea that general arousal improves judgment of attractiveness in men.

Women gave lower scores to female attractiveness. The same pattern has been observed in previous studies, which is interpreted as intrasexual competition leading women to judge same‐sex targets as less attractive (Luxen & Vijver, 2006). In addition, women's arousal is not related to their judgment of facial attractiveness in men (Hagerman et al., 2017). Our results support the sexual strategy theory in which men and women have different mating preferences. As such, women prefer indirect cues (e.g., social status), whereas men prefer direct cues (Buss, 1998; Buss & Schmitt, 2019). Moreover, the alpha power of low‐arousal men was significantly lower than that of women. One reason for this is that men have higher levels of testosterone than women, which diminish the alpha power (Vogel et al., 1971). Another reason may be that woman are more vulnerable to stress‐induced hyperarousal than men (Bangasser et al., 2019). Furthermore, women with arousal levels higher than those of men did not have significant differences in the scores of facial attractiveness. Only men with high arousal levels gave higher attractiveness scores to target faces, indicating that arousal enhances facial attractiveness specifically in men.

Functional connectivity of alpha oscillations predicted individual preferences for facial attractiveness. Traditionally, researchers have focused on explaining observed behavior by manipulating independent variables, which is helpful in elucidating the nature of cognitive processing and behavioral responses (Han, et al., 2020; Thiruchselvam et al., 2016). In this study, we further used CPM to predict individual attractive preference, which revealed that alpha is a biomarker to predict judgment of facial attractiveness in men, supporting the idea that the misattribution of arousal contributes to an individual's facial attractiveness preference (Marin et al., 2017). The results also expanded the theory of misattribution of arousal in that individual spontaneous arousal, not evoked arousal, was associated with facial attractiveness judgment. Additionally, a combined network of delta bands predicted facial attractiveness ratings in women, which may be due to the motivation based on the intrasexual competition, because delta oscillations are related to the motivation process (Knyazev, 2012). These results suggested that the prediction mechanism is sex specific for observers.

Nevertheless, this study had some limitations. First, we only used faces with middle‐level attractiveness. However, high‐ and low‐attractive faces have a different processing mechanism (Han et al., 2020). Researchers can further investigate whether the results in this study are accurate when using high‐ and low‐attractive faces. Second, male faces were not presented to women in this study. Thus, future studies can explore whether male faces are rated as more attractive when presented to women with high arousal.

In conclusion, an individual's general arousal level plays an important role in the judgment of facial attractiveness, particularly for men. Men with high arousal were more attracted to female faces than men with low arousal or women. Our results suggest that individual biological characteristics (i.e., arousal) contribute to the diversity of social functions.

## CONFLICT OF INTEREST STATEMENT

The authors declare no conflicts of interest.

### PEER REVIEW

The peer review history for this article is available at https://publons.com/publon/10.1002/brb3.3132.

## Data Availability

The data that support the findings of this study are available on request from the corresponding author upon reasonable request.

## References

[brb33132-bib-0001] Bangasser, D. A. , Eck, S. R. , & Ordoñes Sanchez, E. (2019). Sex differences in stress reactivity in arousal and attention systems. Neuropsychopharmacology, 44(1), 129–139. 10.1038/s41386-018-0137-2 30022063 PMC6235989

[brb33132-bib-0002] Barry, R. J. , De Blasio, F. M. , Fogarty, J. S. , & Clarke, A. R. (2020). Natural alpha frequency components in resting EEG and their relation to arousal. Clinical Neurophysiology, 131(1), 205–212. 10.1016/j.clinph.2019.10.018 31812081

[brb33132-bib-0003] Beaton, A. A. , Jones, L. , Benton, D. , & Richards, G. (2022). Judgements of attractiveness of the opposite sex and nostril differences in self‐rated mood: The effects of androstenol. Biological Psychology, 167, 108237. 10.1016/j.biopsycho.2021.108237 34864067

[brb33132-bib-0004] Bech‐Sørensen, J. , & Pollet, T. V. (2016). Sex differences in mate preferences: A replication study, 20 years later. Evolutionary Psychological Science, 2, 171–176. 10.1007/s40806-016-0048-6

[brb33132-bib-0005] Brown, A. D. , & Curhan, J. R. (2013). The polarizing effect of arousal on negotiation. Psychological Science, 24(10), 1928–1935. 10.1177/0956797613480796 23925306

[brb33132-bib-0006] Buss, D. M. (1998). Sexual strategies theory: Historical origins and current status. The Journal of Sex Research, 35(1), 19–31. 10.1080/00224499809551914

[brb33132-bib-0007] Buss, D. M. , & Schmitt, D. P. (2019). Mate preferences and their behavioral manifestations. Annual Review of Psychology, 70(1), 77–110. 10.1146/annurev-psych-010418-103408 30230999

[brb33132-bib-0008] Cai, H. , Zhu, J. , & Yu, Y. (2020). Robust prediction of individual personality from brain functional connectome. Social Cognitive and Affective Neuroscience, 15(3), 359–369. 10.1093/scan/nsaa044 32248238 PMC7235956

[brb33132-bib-0009] Carr, E. W. , Huber, D. E. , Pecher, D. , Zeelenberg, R. , Halberstadt, J. , & Winkielman, P. (2017). The ugliness‐in‐averageness effect: Tempering the warm glow of familiarity. Journal of Personality and Social Psychology, 112(6), 1–26. 10.1037/pspa0000083 28368135

[brb33132-bib-0010] Cave, A. E. , & Barry, R. J. (2021). Sex differences in resting EEG in healthy young adults. International Journal of Psychophysiology, 161, 35–43. 10.1016/j.ijpsycho.2021.01.008 33454318

[brb33132-bib-0011] Cloutier, J. , Heatherton, T. F. , Whalen, P. J. , & Kelley, W. M. (2008). Are attractive people rewarding? Sex differences in the neural substrates of facial attractiveness. Journal of Cognitive Neuroscience, 20(6), 941–951. 10.1162/jocn.2008.20062 18211242 PMC3848031

[brb33132-bib-0012] Deakin, J. F. W. , & Exley, K. A. (1979). Personality and male‐female influences on the EEG alpha rhythm. Biological Psychology, 8(4), 285–290. 10.1016/0301-0511(79)90010-3 486627

[brb33132-bib-0013] Delorme, A. , & Makeig, S. (2004). EEGLAB: An open source toolbox for analysis of single‐trial EEG dynamics including independent component analysis. Journal of Neuroscience Methods, 134(1), 9–21. 10.1016/j.jneumeth.2003.10.009 15102499

[brb33132-bib-0014] Delorme, A. , Sejnowski, T. , & Makeig, S. (2007). Enhanced detection of artifacts in EEG data using higher‐order statistics and independent component analysis. Neuroimage, 34(4), 1443–1449. 10.1016/j.neuroimage.2006.11.004 17188898 PMC2895624

[brb33132-bib-0015] Dutton, D. G. , & Aron, A. (1974). Some evidence for heightened sexual attraction under conditions of high anxiety. Journal of Personality and Social Psychology, 23, 510–517. 10.1037/h0037031 4455773

[brb33132-bib-0016] Fayolle, S. , Gil, S. , & Droit‐Volet, S. (2015). Fear and time: Fear speeds up the internal clock. Behavioural Processes, 120, 135–140. 10.1016/j.beproc.2015.09.014 26440426

[brb33132-bib-0017] Fink, A. , & Neubauer, A. C. (2006). EEG alpha oscillations during the performance of verbal creativity tasks: Differential effects of sex and verbal intelligence. International Journal of Psychophysiology, 62(1), 46–53. 10.1016/j.ijpsycho.2006.01.001 16503062

[brb33132-bib-0018] Foster, C. A. , Witcher, B. S. , Campbell, W. K. , & Green, J. D. (1998). Arousal and attraction: Evidence for automatic and controlled processes. Journal of Personality and Social Psychology, 74(1), 86–101. 10.1037/0022-3514.74.1.86

[brb33132-bib-0019] Gao, M. (2020). Connectome‐based models can predict processing speed in older adults. Neuroimage, 223, 117290. 10.1016/j.neuroimage.2020.117290 32871259

[brb33132-bib-0020] Hagerman, S. , Woolard, Z. , Anderson, K. , Tatler, B. W. , & Moore, F. R. (2017). Women's self‐rated attraction to male faces does not correspond with physiological arousal. Scientific Reports, 7(1), 13564. 29051563 10.1038/s41598-017-13812-3 PMC5648837

[brb33132-bib-0021] Han, S. , Hu, J. , Gao, J. , & Fan, J. (2020). Why do you attract me but not others? Retrieval of person knowledge and its generalization bring diverse judgments of facial attractiveness. Social Neuroscience, 15(5), 505–515. 10.1080/17470919.2020.1787223 32602802

[brb33132-bib-0022] Han, S. , Hu, J. , Gao, J. , Fan, J. , Xu, X. , Xu, P. , & Luo, Y. (2022). Comparisons make faces more attractive: An ERP study. Brain and Behavior, 12(6), e2561. 10.1002/brb3.2561 35546305 PMC9226814

[brb33132-bib-0023] Han, S. , Liu, S. , Gan, Y. , Xu, Q. , Xu, P. , Luo, Y. , & Zhang, L. (2020). Repeated exposure makes attractive faces more attractive: Neural responses in facial attractiveness judgement. Neuropsychologia, 139, 107365. 10.1016/j.neuropsychologia.2020.107365 32001231

[brb33132-bib-0024] Hardmeier, M. , Hatz, F. , Bousleiman, H. , Schindler, C. , Stam, C. J. , & Fuhr, P. (2014). Reproducibility of functional connectivity and graph measures based on the phase lag index (PLI) and weighted phase lag index (wPLI) derived from high resolution EEG. PLoS ONE, 9(10), e108648. 10.1002/hbm.25726 25286380 PMC4186758

[brb33132-bib-0025] Härpfer, K. , Spychalski, D. , Kathmann, N. , & Riesel, A. (2021). Diverging patterns of EEG alpha asymmetry in anxious apprehension and anxious arousal. Biological Psychology, 162, 108111. 10.1016/j.biopsycho.2021.108111 33961931

[brb33132-bib-0026] He, D. , Workman, C. I. , He, X. , & Chatterjee, A. (2022). What is good is beautiful (and what isn't, isn't): How moral character affects perceived facial attractiveness. Psychology of Aesthetics, Creativity, and the Arts. 10.1037/aca0000454

[brb33132-bib-0027] Ibrahim, K. , Noble, S. , He, G. , Lacadie, C. , Crowley, M. , McCarthy, G. , Scheinost, D. , & Sukhodolsky, D. (2022). Large‐scale functional brain networks of maladaptive childhood aggression identified by connectome‐based predictive modeling. Molecular Psychiatry, 27(2), 985–999. 10.21203/rs.3.rs-356217/v1 34690348 PMC9035467

[brb33132-bib-0028] Jouffre, S. (2015). Power modulates over‐reliance on false cardiac arousal when judging target attractiveness: The powerful are more centered on their own false arousal than the powerless. Personality and Social Psychology Bulletin, 41(1), 116–126. 10.1177/0146167214559718 25413717

[brb33132-bib-0029] Jozifkova, E. , & Konvicka, M. (2009). Sexual arousal by higher‐ and lower‐ranking partner: Manifestation of a mating strategy? The Journal of Sexual Medicine, 6(12), 3327–3334. 10.1111/j.1743-6109.2009.01526.x 19832934

[brb33132-bib-0030] Klümper, L. , Wühr, P. , & Hassebrauck, M. (2020). Automaticity of facial attractiveness perception and sex‐specific mating strategies. Cognition, 204, 104379. 10.1016/j.cognition.2020.104379 32585470

[brb33132-bib-0053] Knyazev, G. G. (2012). EEG delta oscillations as a correlate of basic homeostatic and motivational processes. Neuroscience & Biobehavioral Reviews, 36(1), 677–695. 22020231 10.1016/j.neubiorev.2011.10.002

[brb33132-bib-0031] Knyazev, G. G. , Savostyanov, A. N. , & Levin, E. A. (2004). Alpha oscillations as a correlate of trait anxiety. International Journal of Psychophysiology, 53(2), 147–160. 10.1016/j.ijpsycho.2004.03.001 15210292

[brb33132-bib-0032] Little, A. C. (2014). Facial attractiveness. Cognitive Science, 5(6), 621–634. 10.1002/wcs.1316 26308869

[brb33132-bib-0033] Luxen, M. F. , & Vijver, F. J. R. (2006). Facial attractiveness, sexual selection, and personnel selection: When evolved preferences matter. Journal of Organizational Behavior, 27(2), 241–255. 10.2307/4093970

[brb33132-bib-0034] Marin, M. M. , & Rathgeber, I. (2022). Darwin's sexual selection hypothesis revisited: Musicality increases sexual attraction in both sexes. Frontiers in Psychology, 13, 971988. 10.3389/fpsyg.2022.971988 36092107 PMC9453251

[brb33132-bib-0035] Marin, M. M. , Schober, R. , Gingras, B. , & Leder, H. (2017). Misattribution of musical arousal increases sexual attraction towards opposite‐sex faces in females. PLoS ONE, 12(9), e0183531. 10.1371/journal.pone.0183531 28892486 PMC5593195

[brb33132-bib-0036] Mengelkoch, S. , Gassen, J. , Prokosch, M. L. , Boehm, G. W. , & Hill, S. E. (2022). More than just a pretty face? The relationship between immune function and perceived facial attractiveness. Proceedings of the Royal Society B: Biological Sciences, 289(1969), 20212476. 10.1098/rspb.2021.2476 PMC8848230 35168398

[brb33132-bib-0037] Montoya, R. M. , & Horton, R. S. (2020). Understanding the attraction process. Social and Personality Psychology Compass, 14(4), e12526. 10.1111/spc3.12526

[brb33132-bib-0038] Oostenveld, R. , Fries, P. , Maris, E. , & Schoffelen, J.‐M. (2011). FieldTrip: Open source software for advanced analysis of MEG, EEG, and invasive electrophysiological data. Computational Intelligence and Neuroscience, 2011, 156869. 10.1155/2011/156869 21253357 PMC3021840

[brb33132-bib-0039] Pandey, G. , & Zayas, V. (2021). What is a face worth? Facial attractiveness biases experience‐based monetary decision‐making. British Journal of Psychology, 112(4), 934–963. 10.1111/bjop.12495 33969477

[brb33132-bib-0040] Pivik, R. T. , & Harman, K. (1995). A reconceptualization of EEG alpha activity as an index of arousal during sleep: All alpha activity is not equal. Journal of Sleep Research, 4(3), 131–137. 10.1111/j.1365-2869.1995.tb00161 10607151

[brb33132-bib-0041] Schwarz, S. , & Hassebrauck, M. (2012). Sex and age differences in mate‐selection preferences. Human Nature, 23(4), 447–466. 10.1007/s12110-012-9152-x 22941269

[brb33132-bib-0042] Shen, X. , Finn, E. S. , Scheinost, D. , Rosenberg, M. D. , Chun, M. M. , Papademetris, X. , & Constable, R. T. (2017). Using connectome‐based predictive modeling to predict individual behavior from brain connectivity. Nature Protocols, 12(3), 506–518. 10.1038/nprot.2016.178 28182017 PMC5526681

[brb33132-bib-0043] Thiruchselvam, R. , Harper, J. , & Homer, A. L. (2016). Beauty is in the belief of the beholder: Cognitive influences on the neural response to facial attractiveness. Social Cognitive and Affective Neuroscience, 11(12), 1999–2008. 10.1093/scan/nsw115 27522090 PMC5141965

[brb33132-bib-0044] Todorov, A. , Olivola, C. Y. , Dotsch, R. , & Mende‐Siedlecki, P. (2015). Social attributions from faces: Determinants, consequences, accuracy, and functional significance. Annual Review of Psychology, 66(1), 519–545. 10.1146/annurev-psych-113011-143831 25196277

[brb33132-bib-0045] Tsukiura, T. , & Cabeza, R. (2011). Shared brain activity for aesthetic and moral judgments: Implications for the Beauty‐is‐Good stereotype. Social Cognitive and Affective Neuroscience, 6(1), 138–148. 10.1093/scan/nsq025 20231177 PMC3023089

[brb33132-bib-0046] van Diessen, E. , Numan, T. , van Dellen, E. , van Lutterveld, R. , & van Dijk, B. W. (2015). Opportunities and methodological challenges in EEG and MEG resting state functional brain network research. Clinical Neurophysiology, 126(8), 1468–1481. 10.1016/j.clinph.2014.11.018 25511636

[brb33132-bib-0047] van Hooff, J. C. , Crawford, H. , & van Vugt, M. (2011). The wandering mind of men: ERP evidence for gender differences in attention bias towards attractive opposite sex faces. Social Cognitive and Affective Neuroscience, 6(4), 477–485. 10.1093/scan/nsq066 20601424 PMC3150857

[brb33132-bib-0048] Vinck, M. , Oostenveld, R. , van Wingerden, M. , Battaglia, F. , & Pennartz, C. M. A. (2011). An improved index of phase‐synchronization for electrophysiological data in the presence of volume‐conduction, noise and sample‐size bias. Neuroimage, 55(4), 1548–1565. 10.1016/j.neuroimage.2011.01.055 21276857

[brb33132-bib-0049] Vogel, W. , Broverman, E. L. , Klaiber, G. , & Cone, F. L. (1971). Effects of testosterone infusions upon EEG of normal male adults. Electroencephalography and Clinical Neurophysiology, 31, 400–403. 10.1016/0013-4694(71)90236-7 4107975

[brb33132-bib-0050] White, G. L. , Fishbein, S. , & Rutsein, J. (1981). Passionate love and the misattribution of arousal. Journal of Personality and Social Psychology, 41(1), 56–62. 10.1002/ejsp.2420110403

[brb33132-bib-0051] Zhou, S. , Li, L. , Wang, F. , & Tian, Y. (2021). How facial attractiveness affects time perception: Increased arousal results in temporal dilation of attractive faces. Frontiers in Psychology, 12, 784099. 10.3389/fpsyg.2021.784099 34956006 PMC8703070

